# Advances in Plant Disease Detection and Monitoring: From Traditional Assays to In-Field Diagnostics

**DOI:** 10.3390/s21062129

**Published:** 2021-03-18

**Authors:** Ilaria Buja, Erika Sabella, Anna Grazia Monteduro, Maria Serena Chiriacò, Luigi De Bellis, Andrea Luvisi, Giuseppe Maruccio

**Affiliations:** 1 Omnics Research Group, Department of Mathematics and Physics “Ennio De Giorgi”, University of Salento, Via per Monteroni, 73100 Lecce, Italy; ilaria.buja@unisalento.it (I.B.); annagrazia.monteduro@unisalento.it (A.G.M.); giuseppe.maruccio@unisalento.it (G.M.); 2 Institute of Nanotechnology, CNR NANOTEC, Via per Monteroni, 73100 Lecce, Italy; mariaserena.chiriaco@nanotec.cnr.it; 3 Department of Biological and Environmental Sciences and Technologies, University of Salento, via Monteroni, 73100 Lecce, Italy; erika.sabella@unisalento.it (E.S.); luigi.debellis@unisalento.it (L.D.B.)

**Keywords:** plant pathogens, environmental monitoring, sensors, IoT and remote sensing

## Abstract

Human activities significantly contribute to worldwide spread of phytopathological adversities. Pathogen-related food losses are today responsible for a reduction in quantity and quality of yield and decrease value and financial returns. As a result, “early detection” in combination with “fast, accurate, and cheap” diagnostics have also become the new mantra in plant pathology, especially for emerging diseases or challenging pathogens that spread thanks to asymptomatic individuals with subtle initial symptoms but are then difficult to face. Furthermore, in a globalized market sensitive to epidemics, innovative tools suitable for field-use represent the new frontier with respect to diagnostic laboratories, ensuring that the instruments and techniques used are suitable for the operational contexts. In this framework, portable systems and interconnection with Internet of Things (IoT) play a pivotal role. Here we review innovative diagnostic methods based on nanotechnologies and new perspectives concerning information and communication technology (ICT) in agriculture, resulting in an improvement in agricultural and rural development and in the ability to revolutionize the concept of “preventive actions”, making the difference in fighting against phytopathogens, all over the world.

## 1. Introduction

**Figure 1:**
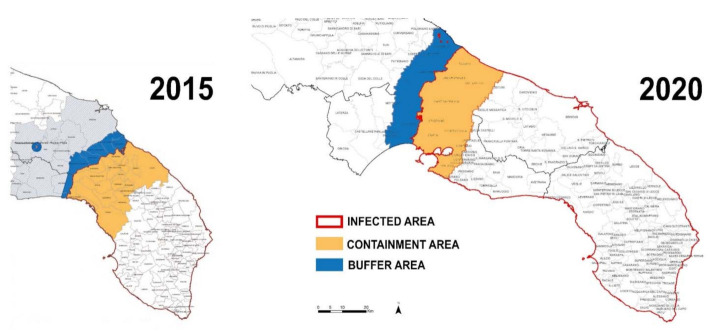
Map showing the spreading of *Xylella fastidiosa* in the Salento peninsula (adapted from [14,15] with permission.).

The search for effective diagnostic tools for plant pathogen detection and management has to face new challenges in an era characterized by climate change and intensified global trades, and recent epidemic events underline its urgency. A large number of harmful “alien” species, such as viruses, phytoplasmas, bacteria, fungi, insects, nematodes, and weeds, travel undisturbed along with people and goods (including plant materials), spreading on a large scale all over the world and causing serious problems to agriculture. Thus, an early detection of plant pathogens is more and more necessary in plant health monitoring in order to manage disease infections in different stages of development, minimizing the risk of disease spreading and avoiding the introduction of new ones [1,2,3,4]. FAO estimates that between 20% and 40% of world crop production is lost annually due to pests, affecting also the main food crops (rice, wheat, corn, potatoes, soy, and cotton) at national and regional level in the different continents [5,6]. Crop losses are correlated with production conditions, having higher losses in food insecure hotspots (with emerging and re-emerging pests/diseases) and lower in those with food surpluses [7]. A top ten list of the main pathogens has been published in ref. [8] and discussed in other works [9,10,11]. Recently the European Commission has also drawn up a list including 20 quarantine organisms, classified as the top priorities for EU Member States, based on their economic, social, and environmental impact: the impinging on crop yield, harm to trade and control costs, unemployment, the step-down of food safety and accessibility, the impact on landscapes, and the reduction of variety in the ecosystem. Over the past decade, the EU has found itself facing several large-scale infectious outbreaks of new plant pests with a significant impact. As an example, the bacterium *Xylella fastidiosa,* also present in A2 list of the European Union and Mediterranean Plant Protection Organization (EPPO) quarantine pathogens [12], represents one of the most dangerous pathogens worldwide, due to the associated severe diseases and its epidemiology. In particular, *X. fastidiosa* subsp. *pauca* strain De Donno is associated with the quick decline syndrome in the olive trees of the Salento peninsula (Italy) [13], with an incredibly fast spread in South Italy (Figure 1) [14,15] and complete destruction of the landscape, aided in this by the meadow bug *Philaenus spumarius* as the main vector. *X. fastidiosa* affects in different ways a wide range of species [16], and, unfortunately, the first symptoms occur several months after the initial infection (“latent period”) [17], facilitating the unnoticed spread of the pathogen. Although widely studied in America, for more than a century, there have been no therapeutic remedies to block the development of the disease, and several genotypes of the bacterium, found in Italy, France, and Spain, represent a serious threat not only to the Mediterranean basin but also to other European regions [18]. As another example, the outbreak triggered by the mushroom *Hymenoscyphus fraxineus*, present for over 20 years in Europe, has caused widespread damage and a high mortality rate in the populations of ash (*Fraxinus excelsior*) without stopping its progress, advancing towards Norway, United Kingdom, Ireland, France, and Italy [19].

International measures are being taken to limit the spread of pests, such as those established by the Commission on Phytosanitary Measures [20] and the very careful phytosanitary surveillance realized by European Food Safety Authority (EFSA) and EPPO. However, there’s much more to be done on the diagnostic front to block, in the bud, the spread of pathogenic organisms, worldwide. One solution could be the development of portable devices, for the simultaneous detection of different phytopathogens, satisfying criteria such as fast response, heterogeneity and complexity of analysis, and ease of use [21]. Reduction of analysis costs is another important parameter, considering the high number of plants commonly involved in monitoring programs. The development of on-field molecular techniques would significantly reduce decision times, decreasing the transmission of pathogens to other plants or the introduction into new geographical areas. In this review, we will first summarize the most widely used techniques in plant diagnostics and then focus on new sensors capable of revolutionizing the approach in the phytopathological field.

## 2. Global Regulatory Framework and Current Methodologies for Fighting against Epidemics

New EU rules are issued to prevent the spread of pests in plants, and to stem potential outbreaks. For example, the Regulation (EU) 2016/2031 [22] concerns protective measures for the preservation of forests and landscapes, with reduced need for pesticides, simplifying documentation for growers and farmers, and providing financial support for surveillance, eradication, and containment. Concerning the globalization of trade, the regulation establishes action to work out the danger posed by these pests and to scale back the risks to a suitable level through phytosanitary measures. Key points regard quarantining pests with criteria to be identified, priority pests, imports and plant passports, and phytosanitary certificates. Accordingly, Regulation (EU) 2017/625 [23] provides rules for farmers, breeders, and traders of plants.

Point-of-care (POC) diagnostics tools are required for their ability to perform analysis and provide prompt responses outside the laboratory, in order to achieve early diagnosis, match surveillance purposes, and prevent large production losses. This is a well-known concept in the human health context [24] with specific criteria described by the World Health Organization under the acronym “ASSURED”: Affordable, Sensitive, Specific, User-friendly, Rapid and Robust, Equipment-free, and Deliverable [25]. The importance of POC also results in the predicted growth of the diagnostics market from US$28.5 billion in 2019 to US$46.7 billion in 2024, at the compound annual growth rate of 10.4% from 2019 to 2024 [26]. POC testing will make the difference in term of management decision, clarifying when to start treatment or to require a confirmation test [27]. POC can support farm animal monitoring, health management [28], and plant pathogen identification [29], in taking rapid disease control measures.

First approaches to prevent plant diseases spreading and implementing quarantine regulations include a visual interpretation of the symptoms and the study of the morphological characteristics of the pathogens through their growth on specific culture media (if cultivable), or through observation with microscopy techniques (if not cultivable in vitro) [30]. Despite their simplicity and low cost, poor reliability and applicability are general problems connected to these techniques, especially in the case of pathogens that are non-cultivable in vitro or difficult to observe under a microscope. In this last case, nucleic acid technology represents the best choice due to rapidity and reliability in diagnosis, with the possibility of analyzing a large number of samples, with high specificity and sensitivity. Classically, in addition to molecular analysis, also serological methods are employed for high-throughput analysis. All these kinds of approaches that regard phenotypic, serological, and molecular techniques are outlined in the EPPO protocols due to the complementary information achievable from several methods [31,32,33]. In Table 1 [34], a list of direct diagnostic methods (able to detect the properties of the pathogen itself) is reported, also summarizing their main features.

Despite the advantages that each technique offers (sensitivity, validation, reliability of results), they require long execution times, bulky instruments, specialized staff, and high costs and may offer late diagnosis. Instead, POC platforms are low-cost, easy to use, smart, and capable of working with small sample volumes, especially after the recent advances in microfluidics [35] offering new solutions also in terms of cloud-connection and smartphone-enabled biosensing [36,37]. Considering the new regulatory requirements and the increasingly stringent and frequent analysis, innovative and rapid techniques, such as POC, must be investigated and developed to carry out a strategic control of the quarantine pathogens and their spreading.

## 3. Innovative Technologies for Plant Pathology

### 3.1. Sensors Platforms for On-Field Monitoring

The need for rapid, low cost, and easy to use technologies has driven the development of various sensors’ platforms enabling a label-free detection of the target pathogens with high sensitivity and specificity, overcoming the limits of traditional diagnosis procedures and the requirement of skilled scientists. An example of label-free detection is given by the use of a quartz crystal microbalance (QCM) to implement an immunosensor for the detection of Maize chlorotic mottle virus (MCMV) [38], as a variation in the resonance frequency of the crystal due to a mass change [39]. Specifically, the authors employed a self-assembled monolayer (SAM) with antibodies specific to MCMV and achieved a detection limit of 250 ng mL^−1^. The sensitivity of this sensor is similar to ELISA test, but it provides other advantages such as simple operation, low cost, rapidity, high sensitivity, and real-time application capability [40]. As another example, Lin et al. [41] exploited surface plasmon resonance (SPR) for a label-free detection monitoring changes in the refractive index on the sensor surface due to the interaction between the analyte in solution and an immobilized ligand. In particular, they used gold nanorods (AuNRs) functionalized with antibodies specific for two orchid’s viruses, Cymbidium mosaic virus (CymMV) or Odontoglossum ringspot virus (ORSV), achieving limits of detection, respectively, of 48 and 42 pg mL^−1^, well below the 1200 pg mL^−1^ value reported by ELISA for both viruses. Another relevant detection strategy is based on surface-enhanced Raman spectroscopy (SERS) for its ability to recognize molecular-fingerprints. A rapid detection of Alternaria mycotoxins in pear fruit, with a LOD of 1.30 μg/L, was demonstrated, using this technique and silver nanoparticles (AgNPs) [42]. Compared to traditional *high-performance liquid chromatography* (HPLC), SERS showed accuracy, high sensitivity, speed, and low LOD.

**Figure 2:**
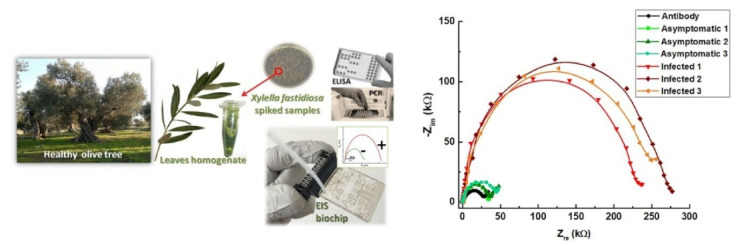
A LOC device for *Xylella fastidiosa* detection in olive trees, exhibiting large variation of EIS signals between asymptomatic trees (reporting impedance values close to the antibody baseline, around 30 kΩ) and symptomatic infected trees (resulting in a range above 200 kΩ) (adapted from [48]—licensed under the Creative Commons Attribution).

In this framework, electrochemical impedance spectroscopy (EIS) sensors [43] are also valuable for plant and crop sciences, e.g., for the detection of plant viruses or pathogens [44]. Beyond being simple and sensitive, EIS is an advantageous technique for on-field analysis because of its ability to provide fast responses without destroying the sample and the availability of portable readers able to monitor changes in the device impedance upon the specific recognition of the target analytes. A DNA hybridization sensor, based on screen-printed carbon electrodes modified with gold nanoparticles (AuNPs), was reported for the selective detection of Citrus tristeza virus (CTV), even in the presence of other non-specific DNAs [45], a feature particularly useful in the case of mixed infections, a quite common situation for cultivated plants. Instead, a selective electrochemical immunosensor was developed, for the detection of Plum pox virus (PPV), using colloidal gold nanoparticles for antibody immobilization, in extracts from plum (*Prunus domestica*) and tobacco (*Nicotiana benthamiana*) leaves [46]. This sensor was capable of discriminating between healthy plants samples and those containing 0.01% of extract from infected plant material, with a very good detection limit of 10 pg mL^−1^ and a dynamic range from 10 to 200 pg mL^−1^ of virus. An evolution of this platform was elaborated then by the same group for the detection of Prunus necrotic ringspot virus (PNRSV) using glassy carbon electrodes as platforms and transducers [47]. Besides viruses, it is also possible to detect bacteria. In this respect, an electrochemical impedance biochip able to discriminate the presence/absence of *X. fastidiosa* subsp. *pauca* strain De Donno in naturally infected olive trees and asymptomatic trees was recently reported [48] (Figure 2). Exhibiting intermediate sensitivity between ELISA and qPCR, this kind of technology could pave the way to monitoring and screening olive trees on field.

**Figure 3:**
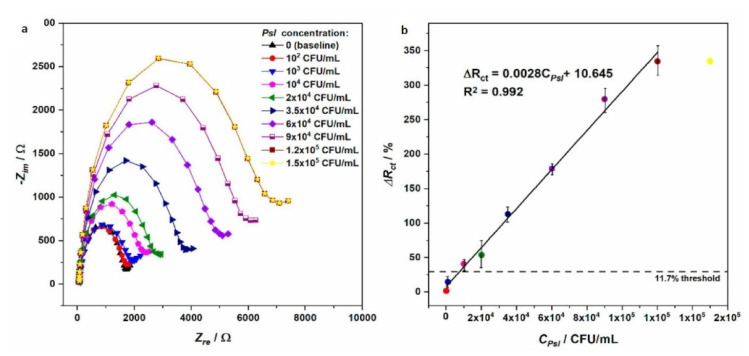
(**a**) Impedance changes obtained with Au/4-ATP/GA/anti-Psl/BSA electrodes, in the case of different concentrations of Psl; (**b**) linear relation between charge transfer resistance changes (ΔRct) and bacteria concentrations (on the right) (reproduced from [49], licensed under the Creative Commons Attribution).

More recently, EIS sensors were used to detect up to seven strains of *Pseudomonas syringae pv. lachrymans* (*Psl*), which is mainly responsible for diseases of many cucurbit species, causing considerable yield losses [49] (Figure 3). The detection was possible in the linear range 1 × 10^3^–1.2 × 10^5^ CFU mL^−1^, showing a sensitivity 30 times higher than the loop-mediated isothermal amplification (LAMP) and also the ability to detect *Psl* strains.

According to Council Directive 2008/90/EC [50], marketing requirements, identification and labeling of varieties, and control measures must be satisfied to ensure buyers receive healthy and good quality propagating materials and fruit plants. The list of the genera and species to which the directive is applied includes *Citrus* spp., *Prunus* spp., and hosts of some *X. fastidiosa* subspecies, among other dangerous pathogens. The intention of the directive is clearly evident in ensuring a widespread fight against the spread of parasites, also with on-the-spot inspections and controls and related marketing ban actions in the event of positivity. Thus, evidences on EIS applications for detection of some widespread pathogens seem to provide interesting perspective and other sectors could also benefit from it, such as the vine, considering the importance of the relevant legislation ([51,52]).

### 3.2. Volatile Organic Compounds Analysis for Pathogen Detection

**Figure 4:**
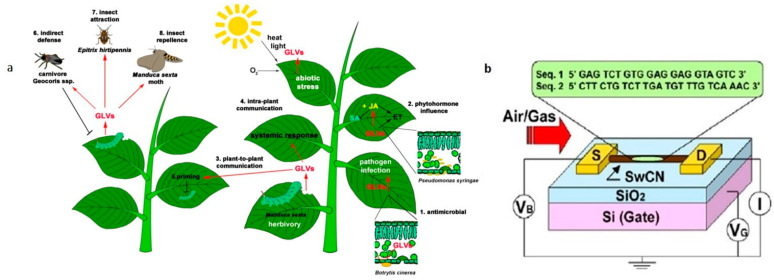
(**a**) Schematic representation of green leaf volatiles (GLVs) emission during herbivory, pathogen infection, and abiotic stress (reproduced with permission from [53], licensed under the Creative Commons Attribution). (**b**) Illustration of the experimental setup made by single-stranded DNA (ss-DNA) and single-walled carbon nanotube field effect transistors (swCN-FETs) (reproduced from [74] with permission.).

The analysis of volatile organic compounds (VOCs) represents an indirect method for plant pathogen detection, since these chemicals are produced by plants, and released as defense mechanism against pathogen attack [53] (Figure 4a). Plant VOCs are characterized as biomolecules and metabolites with high vapor pressure, low boiling point, and low molecular weight. Plants emit many VOCs that serve essential functions in growth, defense, survival, and communication [54], and the pathogen infections of plants could result in the release of specific VOCs, indicative of their physiological health status and thus available for non-invasive monitoring of plant disease. As a result, VOC profiling is emerging as a valuable, non-destructive, rapid tool for plant pathogen detection with good sensitivity and no need of chemical reagents. Traditionally VOCs are detected through gas chromatography–mass spectrometry (GC–MS)-based methods [55,56,57,58], which are complex, time-consuming, expensive, bulky, and require a considerable training for correct use [59]. Recent progresses for VOC’s monitoring are well described in literature [34,60,61,62]. Among alternative, quick, and easy methods, the electronic nose (EN) seems to be a suitable approach for VOCs detection. Compared with GC-MS techniques, EN is a non-invasive, rapid, and cost-effective option for several applications [63]. Introduced to imitate functions of the human olfaction [64], it consists of a multisensory array, an information-processing unit (as an artificial neural network), a software with digital pattern-recognition algorithms, and reference-library databases [65]. Despite the wide range of applications, from agriculture and forestry [66], including plant pest monitoring [67], to food quality [68,69] and the automotive field [70], this technology still has some drawbacks such as the difficulty of detection in open fields due to interference from the surrounding atmosphere, requiring further improvements [71]. Most of the limitations were overcome with the development of a fully automated portable GC device for in situ analysis [59]. The device weighs about 4.5 kg and runs sample collection and analysis autonomously, thanks to a machine learning’s algorithm, developed to evaluate the GC results. The study was conducted on 10 milkweeds (*Asclepias syriaca*) plants, half of which were infested by aphids. Thirty-five VOC peaks were separated and detected in eight minutes, showing a capacity to discriminate between healthy and infected plants with an accuracy of 90–100%, within 48–72 h of attack and 3–4 days earlier if compared to VOC changes detected in other studies.

Another study used a method based on bacteria’s luminescent responses to changes in VOCs [72], in which a whole-cell-based biosensor was developed to detect the presence of the fungus *Penicillium digitatum* in oranges. Specifically, bacterium–alginate beads of *E. coli* strains were placed in a sealed container for an incubation of two hours (with the fruit) and then removed and put into a 96-well-plate for bioluminescence measurements, in a plate reader. On the third day of infection, the four bioluminescent *Escherichia coli* strains allowed detection of fungal activity (before the appearance of visible signs of fungal infection on orange’s surface). This is possible due to the bioluminescent strain’s capability to detect changes in VOC profiles and could encourage the use of bioreporters to be incorporated in field-operable real-time devices, enabling a more efficient postharvest orange management.

Notably, a novel electrical biosensor array based on single-walled carbon nanotubes (SWNTs) functionalized with single-stranded DNA (ssDNA) was recently reported [73] for the detection of four VOCs (ethylhexanol, linalool, tetradecene, and phenylacetaldehyde) compounds, released by Huanglongbing-infected citrus trees, in the asymptomatic phase of the disease. Discrimination of VOCs species and contents was achieved using different mathematical models. The functionalization with ssDNA compensates for the lack of selectivity shown by SWNT-based sensors [74] (Figure 4b), discriminating a variety of odors, with rapid response and recovery times. The ssDNA-SWNT devices showed better sensitivity compared to bare SWNTs, with an excellent reproducibility considering the concentration’s validated range of analytes. However, this technology is still difficult to apply in the field due to lack of specificity, and the authors proposed to focus on mixtures of VOCs emitted by infected citrus trees, in order to make it potentially suitable for real-time detection.

A smartphone-based fingerprinting of leaf volatiles was also recently reported [75] to allow non-invasive diagnosis of late blight, caused by Phytophthora infestans. Plasmonic nanomaterials, used as chemical sensors transducers, targeted green leafy aldehyde, (*E*)-2- hexenal (main late blight marker), down to sub-ppm level of LOD, showing high sensitivity. The system discriminated ten individual plant volatiles, allowing an earlier diagnosis before the manifestation of the symptoms, thanks the aid of an algorithm with a disease detection accuracy above 95%, either in laboratory-inoculated and field-collected tomato leaves. As an indirect method, VOCs can provide an alert about the presence of a disease, but do not identify the responsible pathogen. To respond to online monitoring needs for plant diseases, wearable sensors and Internet of Things (IoT) technologies are presented in the next sections.

### 3.3. Microfluidic-Based Devices for Plant Pathogen Applications

Microfluidics also offers notable opportunities due to the possibility to provide quick and simple sample-in response-out tools, using small sample volumes, and performing sample preparation, reaction, separation, and detection in a single miniaturized system [76]. LOC devices have been rapidly developing since 1980s and early 1990s with different design strategies and techniques [77,78]. Today, microfluidic systems enable rapid identification of chemical and biochemical targets with great versatility [79], spanning from clinical applications [80,81] to environmental monitoring [82,83] and food safety control [84,85]. A common aspect in all cases is the complexity of the samples and, in this respect, microfluidics provides tools able to perform detection overcoming problems due to sample purification and allowing extraction from different matrices through physical, chemical, and biochemical methods [86].

**Figure 5:**
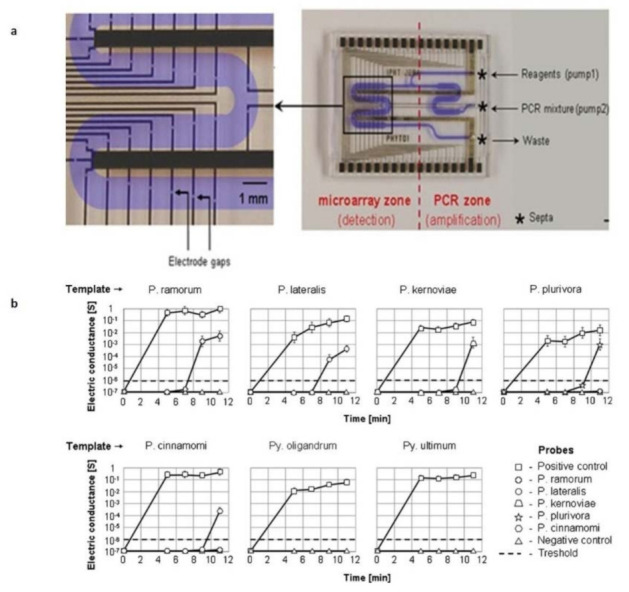
(**a**) A microfluidic chip allowing PCR and hybridization steps; (**b**) Electrical readout, demonstrating detection of five *Phytophthora* species (adapted from [87] with permission).

Considering phytopathology, various applications concern the implementation of miniaturized PCR platforms. For example, an LOC able to perform both PCR and hybridization steps, in 12–15 µl chambers, was developed [87] to detect five *Phytophthora* species with high specificity, with a detection limit of 14.4 fg for target molecule in the PCR mixture (similar to other PCR based methods) (Figure 5a,b). The polymeric microfluidic module seals the two chip components, while leak-proof channels guide the fluids over the microarray zone. Septa allow the sterile injection of fluid samples and reagents, and data were collected through the integration of an electrical readout. A similar approach was used for the detection of selected *Phytophthora* species [88], with the development of a helicase-dependent isothermal amplification (HDA) in combination with on-chip hybridization, employing silver nanoparticles as label and enabling both visual detection and electrical readout. The study showed a successful application in amplifying isolated template DNA from Phytophthora cultures and infected plant material. In another study, an integrated microfluidic system was realized for LAMP detection of *Phalaenopsis orchid* viruses, directly from fresh leaves [89]. The device permitted RNA extraction and purification using magnetic beads, LAMP reaction, and optical (turbidity) detection. The design of the fluidic consists of a reaction chamber where sample, bead hybridization and the RT-LAMP reaction occurred, with three chambers for LAMP reagent storage, one chamber for washing buffer storage, and one positive and negative control chamber for RT-LAMP quality control. Micropumps and microvalves allow automatic fluidic transport and a vacuum pump creates the driving force for fluidic transport. The microfluidic LAMP system showed a detection limit of 35 pg, showing a promising detection system for four orchid viruses: the Cymbidium mosaic virus (CymMV), the Odontoglossum ringspot virus (ORSV), the Capsicum chlorosis virus (CaCV), and the Tomato spotted wild virus (TSWV). A similar integrated LOC was also developed [90]. The improvement of their system consists in a possible use on-field of the device, because it is an automatic system and does not require a bulky gel electrophoresis or fluorescence detection unit. Optical signals caused by the turbidity change, associated with a positive amplification, allowed the detection of RT-LAMP products, directly on the chip, with a limit of detection of 25 fg. This was possible thanks a buried optical fiber-based detection module and a micro-stirring device, integrated into the device. Using an integrated microfluidic system like this, is possible to realize a sensitive, rapid, accurate, and automatic diagnosis of viral pathogens, within only 65 min, as suggested by the authors. Instead, laser-induced fluorescence detection (LIFD) was used for the identification of fungal pathogens DNA [91], combined with a thermal denaturation method, to regenerate the oligonucleotide arrays. More in detail, the fluorophores of hybridized spots inside the microchannel were excited by a green solid state laser during hybridization and denaturation processes, and real-time monitoring was carried out with the aid of a narrow band pass interference filter and a cooled CCD camera used to collect and quantify the fluorescence intensity. In this case, the possible regeneration of the arrays allows a significant reduction of costs and also makes the system promising for the detection of multiple samples, although the use of a cooled CCD camera presents some limitations for field use.

**Figure 6:**
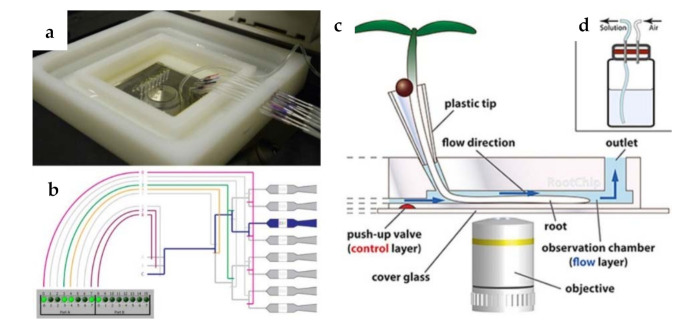
(**a**) Top view of the fully connected RootChip.; (**b**) the valving system and the controller interface; (**c**) image of the RootChip principle; (**d**) image of the pressurizable solution vial with diaphragm (adapted from [97] with permission).

Beyond diagnostic purposes, this kind of technological platforms opens also new avenues for characterizing infection processes that involve bacterial plant pathogens and for recapitulating plants elements/microenvironments such as xylem vessels in order to test new treatments. For example, a microfluidic system was developed to evaluate adhesiveness of bacterial cells (*X. fastidiosa*) to substrata and relate it to type I and/or type IV pili by comparing a wild-type strain with mutants [92]. As shown in this study, microfluidic chambers can provide accurate and easy systems to measure adhesion forces of bacterial cells. The strategy to mimic xylem vessels was also used [93], for the observation of twitching motility by *Xylella*, providing information about bacteria spreading within plants against the prevailing direction of xylem sap. These and other emerging uses of microfluidic systems for food, agriculture, and biosystems industries were reviewed in 2011 in the work of [94], as well as for plant pathology field, as a novel grower-friendly method for plant cells studies [61], including biomechanical investigations [95]. Notably, the Root Chip [96] was able to integrate live-cell imaging of growth and metabolism of *Arabidopsis thaliana* roots with rapid modulation of environmental conditions, allowing an investigation of nutrient uptake in different root zones and the response of individual cells to different environmental stimuli and stress factors. In a following work, [97] (Figure 6a–d) also provided a detailed protocol for studying root processes with this technology, using imaging-based approaches with real time resolution.

### 3.4. Wearable Sensors and Their Support in Real-Time Monitoring

Plants exposed to the field environment undergo different stresses with pathogen infection, such as a combination of drought and heat, drought and cold, salinity and heat, or a combination of these abiotic stresses [98]. Biotic stresses are well described in the scientific literature [99,100,101,102,103]. However, the response of plants to a combination of stresses is unique and cannot be directly extrapolated from their response to each of the different stresses applied individually [98], reducing the reliability of methods that are not based on diagnostic techniques. Recent progress on smart sensor technologies can be exploited to monitor and control important environmental and botanical aspects of plants (abiotic or biotic stress) or plant physiology through a detailed communication of plant health by means of wearable sensors. These offer several advantages, such as helping farm management in the frame of smart agriculture or guiding botanists to understand growth needs.

To accomplish these tasks, sensors must be sufficiently small, compliant, and light enough to not damage or disturb plants in their physiological processes, guaranteeing flexibility, stretchability, and biocompatibility. An example of wearable sensors, with high flexibility, is reported in ref. [104], where SWCNT and graphitic electrodes were prepared onto a variety of both planar and nonplanar substrates, to interface inherent live plants for wireless real-time monitoring of toxic gases. Stomatal electro-mechanical pore size sensors (SEMPSS) were instead developed to trace single stoma-aperture dynamics (stomatal opening and closing latencies) by microscale printing of biocompatible microcircuits directly on the leaf and measuring electrical resistances [105].

**Figure 7:**
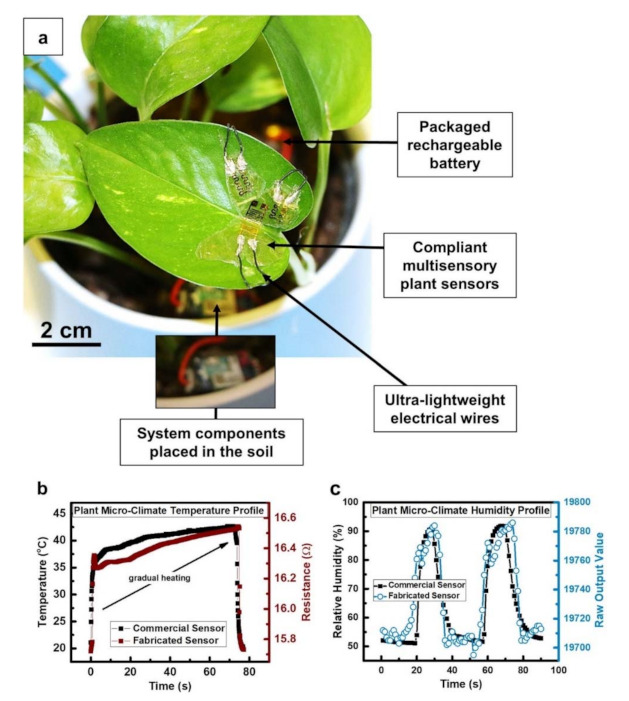
(**a**) Wearable sensors for real-time microclimate monitoring of temperature and humidity levels; (**b**) comparison between real-time response of fabricated and commercial temperature sensors, concerning temperature profile around the plant.; (**c**) real-time plot of fabricated humidity sensor in comparison to a commercial sensor’s behavior, concerning humidity levels around the plant (reproduced from [107], licensed under the Creative Commons Attribution).

Through wearable technologies, monitoring plant growth is also possible, for example, using a simple deposition of graphite/CNT inks to achieve both mechanical stability and stretchability in electrodes, capable of nanometer scale resolution in monitoring plant growth and showing that the growth rates are rhythmic at the time scale of seconds. With this detection, the destruction of plants in pre-treatment phase is also avoided, if compared with traditionally method as scanning electron microscopies [106]. To continuously evaluate optimal growth settings, the effect of the surrounding environment on plants health was also monitored through wearable sensors [107] (Figure 7), collecting information about temperature, humidity, and strain (this latter to monitor plant elongation and growth, with micrometer-level length variations sensitivity and enough stretchability). A biomimetic textile-based biosensor was reported in ref. [108], which can be inserted into plant tissues to monitor variations in the solute content of the sap. Thanks to such sensors, it is also possible to record information about water use for both researchers and farmers. Another interesting application regarded the development of a “plant tattoo sensor”: a tiny graphene sensor that can be taped to plants [109]. The method involves drop-casting a graphene film on polydimethylsiloxane, applying scotch tape to remove the excess graphene from the nonpatterned areas, and then transferring the patterned graphene from the inside of the negative features (channels or cut out areas at the PDMS surface) onto a target tape.

Tiny wearable plant sensors can detect transpiration from plants, without affecting plant growth or crop production. This technology could open a new route in environmental monitoring although, at the moment, its use seems still limited at understanding some physiological responses of the plant (water, nutrients, light, etc.) as well as supporting biomonitoring. On the other hand, the plant’s response is often poorly specific in terms of physiological activity, resulting in only a possible generic stress condition, more complicated to analyze when the stress factors are more than one and of different orders, abiotic and biotic. However, new scientific advances, testing crops for diseases or pesticides, could be also supported with this kind of technology.

### 3.5. IoT and Remote Sensing Technologies

The modern agricultural sector and the food industry are facing challenges such as population growth, climate change, and emerging phytopathological adversities. In this respect, the application of nanotechnologies and Internet of Things (IoT) can contribute significantly, pursuing sustainability [110]. In particular, “real-time communication” and “wireless sensing” are modern concepts in agricultural innovations, with the term “smart farming” describing the application of modern information and communication technologies (ICT) such as remote sensing [111], cloud [112], and Internet of Things (IoT) [113] to help farmers to monitor field conditions from anywhere or with in-field high-tech support. Among them, it is worth also mentioning the aid given by robotics especially concerning seedling and plant management, fruits harvesting, plant protection, or weed control [114].

The concept of remote sensing regards the acquisition of qualitative and quantitative information about an object or environment placed at a distance from a sensor (a satellite, an aircraft, an UAV/UGV, or a probe). For our purposes, how agricultural systems vary in space and time and how this kind of spatially and temporally non-destructive sensing can help in reducing environmental negative impacts by minimizing the resource depletion are important. In particular, it is possible to analyze molecular interactions and crop stress and its biophysical or biochemical characteristics [115], as well as to detect (even at early stages) variations induced in plants under stress conditions (leaf area index, chlorophyll content, or surface temperature), generating a different fingerprinting compared to the healthy condition [116]. The use of remote sensing in precision farming applications started in the 1980s and initially regarded only few visible or near infrared bands, but was then further developed as hyperspectral remote sensing. As well summarized in another work [117], plant-related events can be monitored in different spectral regions: pathogen propagules in the VIS (depending on the pathogen); chlorophyll degradation (necrotic or chlorotic lesions) in the VIS and red-edge (550 nm; 650–720 nm); photosynthesis disturbance as fluorescence (450–550 nm; 690–740 nm) and in the TIR (8000–14,000 nm); senescence in the VIS and NIR (680–800 nm) due to browning and SWIR (1400–1600 nm and 1900–2100 nm) due to dryness; changes in canopy density and leaf area in the NIR; and changes in the transpiration rate in the TIR (8000–14,000 nm). Clearly, it is possible to use them to detect the presence of disease in field crops. Recently, a hyperspectral radiometer was used to estimate (from leaf reflectance) the intrinsic efficiency of photosystem II photochemistry, namely the ratio Fv/Fm among two leaf ChlF-derived parameters, which represent, respectively, the variable and the maximum fluorescence [118]. In stressed leaves, Fv/Fm dramatically dropped (leaf chlorophyll content remained unchanged). In parallel with this decrease, the slope of reflectance in the spectral range 700–900 nm was observed to increase, with high correlation of the first derivative reflectance in the NIR regions with Fv/Fm.

An aid can also be offered by agricultural drones also known as UAVs (unmanned aerial vehicles), which can offer support in surveillance activity, helping human activities such as planting crops, fighting pests, and crop monitoring. For example, the “Sense Fly” [119] agriculture drone eBee SQ uses multispectral image analyses, communicating with eMotion Ag software. Among the characteristics, the software supports the direct uploading of the drone’s multispectral images to cloud services, covering hundreds of acres, for an accurate crop monitoring and analysis. The use of aircraft or satellites technologies is instead well described [120,121]. In particular, the former offers an overview about remote sensors on satellites and aircraft, considering also agriculture applications (data of landsat and GIS concerning land use and nitrogen flow, the use of aerial hyperspatial data for wheat growth estimation or farmland analysis and Aerial Lidar Data for 3-D remote sensing for terrain and forests). The latter focuses the attention on benefits and limits of satellites, UAS, and ground sensors, underlining UAS versatility or the suitability of the two other systems for specific applications (as on-the-go processing capabilities, for some ground sensors, allowing instant herbicide applications, without data processing delays). An evolution of this technology regards low-cost mini-UAV for thermal- and multispectral-imaging, as described in ref. [122]. In this study the authors used a mini-UAV system (HiSystems’ MK-Okto), which has a payload of approximately 1 Kg, sufficient to be equipped with a handheld low-weight NEC F30IS thermal imaging system and a four band multispectral imaging system (Tetracam’s Mini MCA). The system was demonstrated to be useful for the acquisition of thermal and multispectral images, ensuring comparability of the data thanks to georeferencing. The time flight of 15 min allows for small scale applications.

Other application of remote sensing concerns agricultural land use monitoring, crop yield forecasting, monitoring crops for yield optimization, and ecosystem services [123]. Additionally, an overview on remote sensing for environmental monitoring is well described [124], which explains Earth’s surface monitoring and characterization, providing also information on ecosystem sustainability, drought mitigation, human health, and other environmental studies.

**Figure 8:**
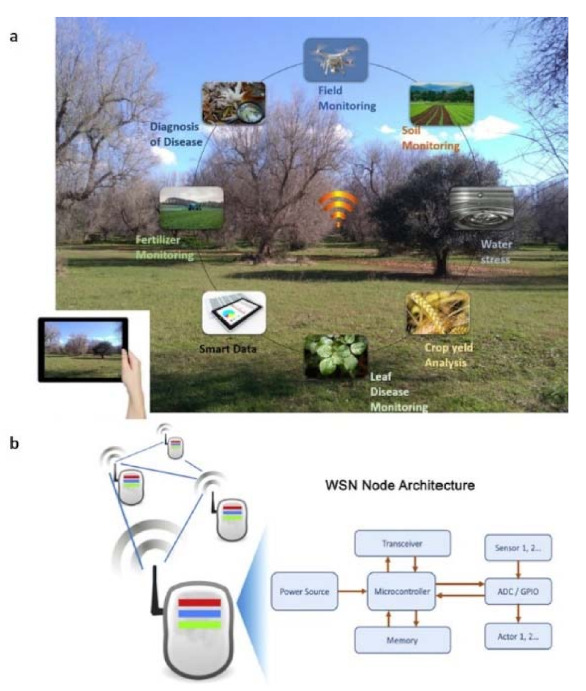
(**a**) Panoramic representation of IoT applications in agriculture field. (**b**) The architecture of a wireless sensor node consists, schematically, of a processing module, one or more sensor modules, and an RF communication module (reproduced from [128] with permission.).

IoT technology is becoming increasingly popular for its several fields of application, as an emerging technology based on connectivity. The term was first coined by Kevin Ashton in 1999 in the context of supply chain management [125] and offers many innovative solutions, with technologies such as Radio Frequency Identification (RFID) [126], wireless sensor networks, and cloud computing, in different areas such as healthcare, retail, traffic, security, smart homes, smart cities, and agriculture [127]. The IoT technologies are mainly based on three building blocks: (i) a sensing component, (ii) data transfer (that functions as a network) and data storage, and (iii) a manipulation component. [128]. The IoT scenario is still developing [129,130,131] due to continuous technological advances, even in the agriculture field (Figure 8a) in order to help farmers or institutions to manage early or preventive actions to fight phytopathogens. Some relevant applications of IoT in agriculture are described in ref. [132,133,134,135]. In particular, a system based on wireless sensors was reported in ref. [135] as a crop monitoring network, enabling the gathering of data concerning temperature, humidity, and also crop growth images, through which it is possible to observe crops intuitively and distinctly. Systems based on wireless sensors are even adopted in blueberry planting areas [136]. Instead, thanks to the aid of a solar energy supply system, in ref. [137], the ability of the sensor nodes to collect parameters such as temperature, humidity of the greenhouse, carbon dioxide content, and intensity of illumination was shown. In Figure 8b, a schematic architecture of a typical wireless sensor node is shown.

Advantages of IoT include, for example, the possibility to support sanitary certification or achieving production data to support traceability. Radiofrequency identification (RFID) microchips were implanted for identifying, storing, and tracking *Prunus* spp. plants [138], as well as supporting clonal selection of grapevine [139]. In this latter, RFID technology has been also successfully used to identify all plants during ampelographic, genetic, and sanitary checks. RFID potentialities consists also in providing a system to retrieve propagated material [140], tagging basic material to establish mother plant vineyards and derived certified material. Concerning the careful management of phytosanitary treatments, it is possible to detect pests as the borer insects in tomatoes, using video processing, cloud computing, and robotics [141]. A real-time video of tomato crops is captured and sent to an application hosted on cloud for processing. Based on image analysis results, a robot is instructed to spray pesticides with a fully automated method able to perform a constant surveillance of the farm. A wireless sensor network with autonomous and self-powered nodes deployed throughout a vineyard was also proposed [142]. The hardware and software platform VineSens is able to prevent diseases like downy mildew, thanks to the use of epidemiological models, helping farmers in management and enabling substantial savings, e.g., to decrease the amount of phytosanitary treatments. Through the platform, it is also possible to collect weather data from different spots of the vineyard, and the access to them is guaranteed through a web-based interface using desktop or mobile devices. Monitoring tools for crop management and detection of insect pests also include use of remote sensed imagery and geospatial image processing through unmanned aerial vehicles (UAVs) [143], where the methodology uses an integrated UAV with advanced hyperspectral, multispectral, and digital RGB sensors combined with terrain-based data. Crop monitoring also includes systems like “Arable” and “Semios”. The former communicates weather and plant measurements to the cloud, providing a continuous visibility of stress, pest, and disease indicators and giving the access to data through a software platform anywhere, in real time [144]. The letter uses a patented mesh network that manages each orchard block, providing the installation of remote-controlled pheromone dispensers, camera traps for pests, soil moisture sensors, or leaf-wetness devices [145].

Pathogens monitoring, as that realized for *Xylella fastidiosa* (*Xf*) [146], can be actuated using airborne platforms carrying multispectral and thermal cameras, selecting spectral bands for their sensitivity to the Xf symptoms (precisely, blue bands coupled with thermal region). In another work [147], a 3D radiative transfer modelling approach (3D-RTM) was developed, integrating airborne hyperspectral imagery and Sentinel-2 satellite data, to assess spatio-temporal dynamics of *Xf* infections in olive orchards. Results showed that Sentinel-2 time-series imagery could provide useful spatio-temporal indicators to monitor the damage caused by Xf infections across large areas. Other examples of agricultural applications are well described [148,149,150] and a review on current studies and research works in agriculture concerning big data analysis is given [151]. In terms of exploiting nanotechnology, another study [152] described how miniature sensors, interconnected through nano-networks, could obtain fine-grained data within objects and from hard-to-access areas. A thorough understanding of the topic is given [153] and includes architectures, domains, trends, possibilities, and challenges.

## 4. Discussion and Future Perspectives

**Figure 9:**
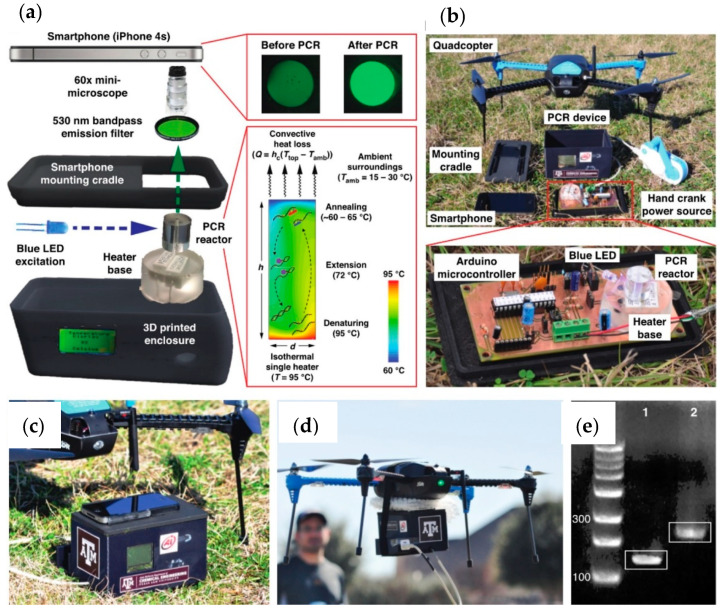
Lab-on-a-drone components: (**a**) the convective thermocycling with a single heater, for PCR reactions; (**b**) instrument assembly with available components; (**c**) A smartphone camera is used for fluorescence detection of reaction products; (**d**) the entire assembly, characterized by lightweight; (**e**) successful in-flight replication of two different DNA targets. (Reproduced from [154] with permission; further permissions related to the material excerpted should be directed to the ACS).

Existing techniques for detection of plant diseases have been reviewed, and an overview of innovative methods enabling identification of symptoms and preventive actions against pathogens spreading has been provided. We also summarized progresses related to sensors and microfluidics technologies, considering recent advances also in wearable sensing and IoT technologies. Today, new perspectives are emerging, thanks to the combination of various bio-sensing platforms within smartphone-integrated electronic readers [36]. An interesting perspective concerns the integration of skin-like flexible sensors with wireless communication technology for real-time plants monitoring. New “lab-on-a-drone” analysis platforms can instead result from a combination of sensing and robotics technologies, allowing rapid in-flight assays with smartphone connectivity, eliminating waste of time due to sample collection and analysis, and enabling emergency response, agricultural bio-surveillance, and veterinary field care scenarios. For example, using consumer-class quadcopter drone with smartphone connectivity (Figure 9), for in-field nucleic acid-based diagnostics, Priye et al. [154] demonstrated flight replication of *Staphylococcus aureus* and λ-phage DNA targets in less than 20 min. Smartphone technology can also contribute to more accurate, smart, and portable diagnosis systems [155,156] in which connectivity, high-quality images, and processing capacity of these devices could help farmers and institutions in fighting plant diseases, all over the world. All these technologies are able to communicate to each other and open new avenues for fighting plant pathogens and their spreading, in an efficient and intuitive way.

**Figure 10:**
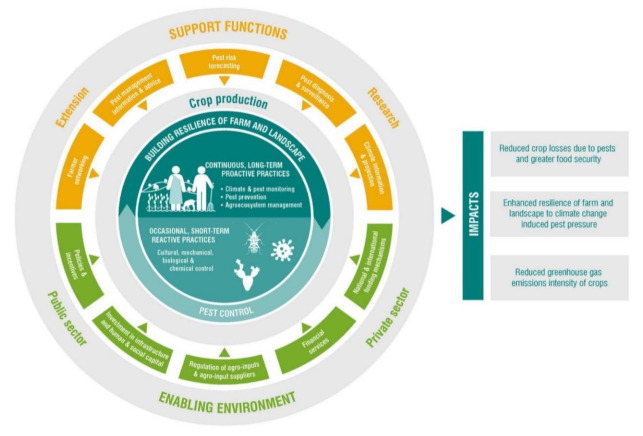
Schematic representation of interdisciplinary approaches and strategies for Climate-Smart Pest Management (CSPM) and its impact. (Reproduced from [157]—licensed under the Creative Commons Attribution).

In addition to the mentioned technological progresses, interdisciplinary approaches such as the Climate-Smart Pest Management (CSPM) are also becoming available, with the implementation of holistic strategies that includes farmers, extension workers, researchers, and public and private sector stakeholders, acting in synergy to increase resilience of farms and landscapes (from changing pest threats to food security, [157]) (Figure 10). This approach can overcome various limitations due to a strict interconnection between research and the public/private sector.

## 5. Conclusions

Several opportunities are today enabled by technological innovation in the field of plant diagnostics, and EPPO diagnostic protocols are being updated. However, there are still challenges in making new approaches available on a large scale, if compared to other areas of manufacturing. In this respect, it is worth noting that not only can preexisting and smart technologies help scientists in fighting pandemic diseases and spreading of unknown pathogens, but the cooperation among heterogeneous scientific groups, public and private sector stakeholders, and farmers can also make the difference.

## Figures and Tables

**Table 1:** Comparison of main techniques for the detection of plant diseases and their characteristics. PCR: polymerase chain reaction; FISH: fluorescence in-situ hybridization; ELISA: enzyme-linked immunosorbent assay; IF: immunofluorescence; FCM: flow cytometry; CFU: colony forming unit (adapted from [34]—licensed under the Creative Commons Attribution).

Techniques	Limit of Detection (CFU/mL)	Advantages	Limitations
PCR	10^3^–10^4^	Mature and common technology, portable, easy to operate	Effectiveness is subjected to DNA extraction, inhibitors, polymerase activity, concentration of PCR buffer, and deoxynucleoside triphosphate
FISH	10^3^	High sensitivity	Autofluorescence, photobleaching
ELISA	10^5^–10^6^	Low cost, visual color change can be used for detection	Low sensitivity for bacteria
IF	10^3^	High sensitivity, target distribution can be visualized	Photobleaching
FCM	10^4^	Simultaneous measurement of several parameters, rapid detection	High cost, overwhelming unnecessary information

## Data Availability

Not applicable.
